# Bacterial profile and antibiotic susceptibility of bloodstream infections among infants, children, and adolescents undergoing treatment for acute leukemia

**DOI:** 10.1371/journal.pone.0358779

**Published:** 2026-09-24

**Authors:** Razan Zatarah, Tala Albdour, Alaa Assad, Rawan Budair, Lama Nazer

**Affiliations:** 1 Department of Pharmacy, Clinical Pharmacy Section, King Hussein Cancer Center, Amman, Jordan; 2 Department of Pediatrics, Pediatric Hematology & Oncology/ Infectious Disease Section, King Hussein Cancer Center, Amman, Jordan; Aga Khan University, PAKISTAN

## Abstract

**Introduction:**

Pediatric patients with acute leukemia are at risk of bacteremia due to disease and treatment-related toxicities. Limited data exists on pathogen distribution across different pediatric age groups. This study aims to investigate the distribution of bacterial pathogens and their susceptibility patterns across infants, children, and adolescents undergoing treatment for acute leukemia.

**Methods:**

Retrospective cohort study that included pediatric patients with Acute Lymphoblastic Leukemia (ALL) or Acute Myeloid Leukemia (AML) who had positive blood cultures between 01/01/2015–31/12/2024. Bacteremia was defined as the presence of bacterial microorganisms in the bloodstream, confirmed by one or more positive blood cultures with associated symptoms. Patients were categorized into three age groups: infants (≤1 year), children (>1 to <12 years), and adolescents (≥12 to ≤18 years). Collected data included demographics, leukemia disease risk (high, standard or low), treatment phase (induction, consolidation, maintenance, relapse, or palliative care), signs and symptoms and the causative pathogen along with its susceptibility and resistance testing. Descriptive statistics were used to summarize findings.

**Results:**

A total of 308 bacteremia episodes among 160 patients were identified. Of these, 291 (94%) episodes occurred in ALL patients and 17 (6%) in AML patients. The median age was 5 years (IQR 3–11 years) with children, adolescents, and infants representing 78%, 21%, and 1%, respectively. A total of 330 bacterial isolates were identified. Isolates were most frequently recovered during consolidation (30%), induction (24%), and maintenance (23%) phases. Gram-positive organisms, particularly *Staphylococcus spp*. predominated across all treatment phases and age groups. Whereas a higher proportion of Gram-negative organisms – including *Escherichia coli*, *Klebsiella spp., and Pseudomon*as *spp.* – were observed among adolescents and patients in high-risk groups. Antimicrobial resistance was confined to *Escherichia coli* and *Klebsiella spp*. exhibiting Extended-Spectrum β-lactamase and Carbapenem-Resistant Enterobacterales phenotypes across the cohort.

**Conclusions:**

In this single-center descriptive study, bacteremia episodes and bacterial isolates showed descriptive differences across age groups, treatment phases, and leukemia risk categories. These findings support continued local infection surveillance. Larger multicenter studies with sufficient subgroup representation are needed to determine whether age- or phase-specific empiric therapy strategies are warranted.

## Introduction

Bacteremia remains a significant concern among pediatric patients diagnosed with Acute Lymphoblastic Leukemia (ALL) and Acute Myeloid Leukemia (AML). These patients are highly susceptible to infections due to their immunosuppression resulting from the disease itself or from intensified chemotherapy regimens [1]. Despite advancements in supportive care, bacteremia continues to be a major cause of morbidity and mortality [2]. Understanding the distribution of bacteremia across different pediatric age groups is crucial for developing individualized targeted prevention and treatment strategies.

The general pediatric population is heterogeneous in nature, with variations in infection susceptibility, clinical manifestations, and pathogen distribution across age groups. Infants, for instance, might have different vulnerabilities due to immature immune systems and less developed mucosal barriers compared to older children and adolescents, who may have distinct patterns of exposure and immune responses [3]. Some studies have reported age-dependent variation in bacterial isolates, with *Escherichia coli* becoming increasingly prevalent after 5 years of age and peaking among children aged ≥10 years, while *Klebsiella pneumoniae* has been observed most frequently in the 5–9 years age group [4].

Variations in leukemia subtypes (ALL vs. AML), treatment intensity and treatment phase could further influence the risk of bacteremia [5]. Existing literature reports overall incidence rates or focuses on specific treatment phases, such as induction or consolidation; few have assessed the incidence of bacteremia throughout the entire treatment course or phases with less intensive chemotherapy such as maintenance and palliative care [6–9].

While some studies have reported age-stratified data, they are limited and do not comprehensively assess differences across the full pediatric age spectrum or throughout leukemia treatment. Additionally, data linking age-specific pathogen distribution with antibiotic susceptibility, particularly across key classes such as β-lactams, aminoglycosides and glycopeptides, remain limited. Accordingly, this study aimed to investigate the distribution of bacterial pathogens and their susceptibility profile across different age groups of pediatric patients with acute leukemia throughout the entire course of treatment.

## Materials and methods

This was a single-center retrospective study conducted at King Hussein Cancer Center (KHCC) located in Amman, Jordan. KHCC is a leading hospital in the Middle East region that offers pediatric oncology services, including advanced treatment for pediatric patients with leukemia, lymphoma, and solid tumors. The study was approved by the KHCC institutional review board (24 KHCC 113), with a waiver of consent given the retrospective nature of the study.

We included all pediatric patients with ALL and AML who were diagnosed with bacteremia upon admission or during their hospital stay between 01/01/2015–31/12/2024. Data collection covered this entire period; however, data were accessed and extracted for this research study between 01/07/2024 and 31/12/2024. Patients under the bone marrow transplant service and those who initiated their leukemia treatment prior to being transferred to KHCC were excluded from the study. The electronic medical records were used to identify patients with bacteremia, defined as the presence of viable bacterial microorganisms in the bloodstream evidenced by one or more positive blood cultures with associated clinical symptoms such as documented fever (≥38°C), chills/shivering, tachycardia, hypotension, mucositis [10].

Patients were categorized into three groups based on age: infants (≤1 year), children (>1 to <12 years), and adolescents (≥12 to ≤18 years). The treatment course was defined as the period beginning at initial diagnosis and extending through all planned phases of treatment. For patients who experienced disease relapse or transitioned to palliative care, these phases were also considered part of the treatment course. The following data were collected for each bacteremia episode: gender, type and subtype of acute leukemia, disease risk and treatment phase of acute leukemia as per KHCC guidelines (risk group classified as high, standard, or low; phases as induction, consolidation, maintenance, relapse, or palliative care) [11–15], remission status, central nervous system (CNS) involvement, presence and type of indwelling central venous catheter (CVC), degree of neutropenia based on absolute neutrophils counts (ANC), presenting signs and symptoms and the causative bacterial pathogen along with its susceptibility and resistance testing.

At our center, newly diagnosed pediatric patients with ALL are classified and treated according to the St. Jude Total XV and Total XIII protocols [11,12]. For relapsed ALL, management follows either the UKALLR3 or the St. Jude ALL-R16 protocol, depending on physician preference and patient clinical status [13,14]. Newly diagnosed pediatric patients with AML are treated according to the St. Jude AML02 trial [15]. Infection prophylaxis included oral and perianal care (sodium bicarbonate mouthwash, mycostatin or miconazole oral gel, povidone solution, and zinc oxide cream), along with trimethoprim/sulfamethoxazole administered twice daily on weekends for *Pneumocystis jirovecii* prophylaxis. Patients with contraindications to trimethoprim/sulfamethoxazole received pentamidine or dapsone. In addition, ciprofloxacin and vancomycin prophylaxis were administered to AML patients post-chemotherapy until count recovery, defined as an ANC > 1000 cells/µL [15].

Microbiology results and susceptibility patterns of pathogens towards antibiotics were based on local testing performed at KHCC in accordance with Clinical and Laboratory Standards Institute (CLSI) guidelines. Bacterial isolates were classified as non-susceptible if they showed resistant or intermediate susceptibility [16]. Clinical analyses were performed at the bacteremia episode level, while microbiological analyses were conducted at the bacterial isolate level. Unless otherwise stated, patient-level characteristics are counted at the episode level, and patients with multiple episodes may contribute more than once. Blood cultures that were considered contaminants were excluded. A contaminant was defined as a single positive culture without accompanying clinical signs or symptoms of infection (fever, chills/shivering, tachycardia, hypotension, or mucositis). Polymicrobial cultures obtained within the same clinical infectious episode were counted as one bacteremia episode but multiple bacterial isolates; cultures yielding a different organism after clinical resolution or after a documented negative blood culture were counted as a new bacteremia episode. Duplicate cultures yielding the same organism during the same admission were considered a single bacteremia episode and isolate unless separated by a documented negative blood culture, in which case they were considered a new bacteremia episode and isolate.

Categorical data were presented as counts and percentages while continuous data were presented as median and interquartile range (IQR).

## Results

Over the study period, 308 bacteremia episodes for 160 patients were identified. Of these, 291 (94%) episodes occurred in ALL patients and 17 (6%) in AML patients. The median age was 5 years (IQR 3–11 years) with children, adolescents, and infants representing 78%, 21%, and 1%, respectively. Males accounted for 194 (63%) of the episodes. Across all age groups, bacteremia was most observed during induction, consolidation and maintenance phases combined; while fewer episodes were seen during relapse and palliative care. Table 1 outlines the baseline demographics and clinical characteristics of bacteremia episodes in pediatric patients with acute leukemia.

**Table 1:** Baseline demographics and clinical characteristics of bacteremia episodes in pediatric patients with acute leukemia.

Characteristics		n= 308 bacterial episodes
**Gender**	Male	194 (63%)
	Female	114 (37%)
**Age group**	Infants	2 (1%)
	Children	240 (78%)
	Adolescents	66 (21%)
**Primary Diagnosis**	Acute lymphoblastic leukemia	291 (94%)
	B-cell ALL	264
	T-cell ALL	27
	Acute myeloid leukemia	17 (6%)
	M1	3
	M2	9
	M3	1
	M4	2
	M7	2
**Treatment phase**	Induction	76 (25%)
	Consolidation	91 (30%)
	Maintenance	72 (23%)
	Relapse	57 (19%)
	Palliative	12 (3%)
**Remission status**	Remission	165 (54%)
	Non-remission	143 (46%)
**CNS status**	Blasts	4 (1%)
	No-blasts	304 (99%)
**Mucositis**	Yes	27 (9%)
	No	281 (91%)
**Chills/rigors**	Yes	32 (10%)
	No	276 (90%)
**Fever**	Yes	245 (80%)
	No	63 (20%)
**Tachycardia**	Yes	99 (32%)
	No	209 (68%)
**Hypotension**	Yes	80 (26%)
	No	228 (74%)
**Absolute neutrophil counts**	< 500 cells/µL	179 (58%)
	500-1000 cells/µL	109 (35%)
	> 1000 cells/µL	20 (7%)
**Line access**	Central line	128 (42%)
	Central venous catheter	7
	Hickman catheter	5
	Peripherally Inserted Central Catheter	12
	Port catheter	104
	Peripheral line	180 (58%)

Unless otherwise stated, Table 1 summarizes bacteremia episodes; patient-level characteristics are therefore counted at the episode level, and patients with multiple episodes may contribute more than once.

A total of 330 bacterial isolates were identified, including 312 isolates from patients with ALL (284 B-cell ALL and 28 T-cell ALL) and 18 isolates from patients with AML. Among patients with B-cell ALL, bacterial isolates were most frequent during maintenance (26%), induction (25%), and consolidation (24%). Gram-positive organisms predominated across all treatment phases, particularly among children, whereas a higher proportion of Gram-negative organisms was observed among adolescents during relapse and the palliative care phases. In T-cell ALL, isolates were most common during consolidation (57%). The overall pathogen distribution was broadly comparable to that observed in B-cell ALL, with *Staphylococcus spp.* representing the most frequently isolated organisms. A relatively higher proportion of *Micrococcus spp*. was noted among both children and adolescents. Table 2 presents the distribution of bacterial isolates by age group and treatment phase in pediatric patients with acute leukemia.

**Table 2:** Distribution of bacterial isolates by age group and treatment phase in pediatric patients with acute leukemia.

Diagnosis	Age Group	Treatment phase	Total isolates	Top three organism(s)
B-cell lymphoblastic leukemia	Infants	Induction	2	*Staphylococcus spp. (n=2)*
	Children	Induction	58	*Staphylococcus spp.(n=20)*
				*Escherichia coli (n=4)*
				*Acinetobacter spp. (n=4)*
		Consolidation	56	*Staphylococcus spp. (n=25)*
				*Streptococcus spp. (n=7)*
				*Klebsiella spp. (n=4)*
		Maintenance	65	*Staphylococcus spp. (n=27)*
				*Streptococcus spp. (n=6)*
				*Campylobacter spp. (n=5)*
		Relapsed	45	*Staphylococcus spp. (n=16)*
				*Klebsiella spp. (n=8)*
				*Escherichia coli (n=4)*
		Palliative	12	*Klebsiella spp. (n=5)*
				*Escherichia coli (n=5)*
				*Staphylococcus spp. (n=2)*
	Adolescents	Induction	11	*Klebsiella spp. (n=3)*
				*Pseudomonas spp. (n=3)*
				*Bacillus spp. (n=2)*
		Consolidation	12	*Staphylococcus spp. (n=4)*
				*Pseudomonas spp. (n=3)*
				*Streptococcus spp. (n=2)*
		Maintenance	8	*Staphylococcus spp. (n=4)*
				*Corynebacterium spp. (n=1)*
				*Micrococcus spp. (n=1)*
		Relapsed	14	*Escherichia coli (n=6)*
				*Klebsiella spp. (n=2)*
				*Micrococcus spp. (n=2)*
		Palliative	1	*Corynebacterium spp. (n=1)*
T-cell lymphoblastic leukemia	Infants	___	___	___
	Children	Induction	6	*Staphylococcus spp. (n=4)*
				*Micrococcus spp. (n=1)*
				*Kocuria spp. (n=1)*
		Consolidation	7	*Staphylococcus spp. (n=2)*
				*Klebsiella spp. (n=2)*
				*Anaerococcus spp. (n=2)*
		Relapsed	3	*Anaerococcus spp. (n=1)*
				*Staphylococcus spp. (n=1)*
				*Stenotrophomonas (n=1) Maltophilia*
	Adolescents	Consolidation	9	*Staphylococcus spp. (n=4)*
				*Klebsiella spp. (n=3)*
				*Micrococcus spp. (n=1)*
		Maintenance	3	*Staphylococcus spp. (n=2)*
				*Micrococcus spp. (n=1)*
Acute myeloid leukemia	Infants	___	___	___
	Children	Induction	5	*Staphylococcus spp. (n=4)*
				*Anaerococcus spp. (n=1)*
		Consolidation	4	*Staphylococcus spp. (n=1)*
				*Streptococcus spp. (n=1)*
				*Micrococcus spp. (n=1)*
	Adolescents	Consolidation	9	*Escherichia coli (n=3)*
				*Acinetobacter spp. (n=2)*
				*Staphylococcus spp. (n=2)*

The distribution of bacterial isolates was summarized across leukemia risk categories and age groups. The largest number of bacterial isolates was observed among high-risk patients with ALL and AML across the age groups. *Staphylococcus spp.* were the most frequently observed organisms in most subgroups. Gram-negative organisms, including *Escherichia coli, Pseudomonas spp., and Klebsiella spp.*, were also observed among children and adolescents in the standard- and high-risk groups. Table 3 presents the distribution of bacterial isolates by age group and leukemia risk category.

**Table 3:** Distribution of bacterial isolates by age group and leukemia risk group.

Diagnosis	Age group	Risk group	Total isolates	Top three organism(s)
B-cell lymphoblastic leukemia	Infants	High	2	*Staphylococcus spp. (n=2)*
	Children	Low	59	*Staphylococcus spp. (n=28)*
				*Pseudomonas spp. (n=5)*
				*Streptococcus spp. (n=4)*
		Standard	64	*Staphylococcus spp. (n=20)*
				*Streptococcus spp. (n=8)*
				*Escherichia coli (n=8)*
		High	113	*Staphylococcus spp. (n=40)*
				*Escherichia coli (n=10)*
				*Klebsiella spp. (n=10)*
	Adolescents	Standard	7	*Escherichia coli (n=3)*
				*Pseudomonas spp. (n=1)*
				*Staphylococcus spp. (n=1)*
		High	39	*Staphylococcus spp. (n=8)*
				*Pseudomonas spp. (n=5)*
				*Klebsiella spp. (n=4)*
T-cell lymphoblastic leukemia	Infants	___	___	___
	Children	High	15	*Staphylococcus spp. (n=7)*
				*Anaerococcus spp. (n=3)*
				*Klebsiella spp. (n=2)*
	Adolescents	High	12	*Staphylococcus spp. (n=4)*
				*Klebsiella spp. (n=3)*
				*Micrococcus spp. (n=2)*
Acute myeloid leukemia	Infants	___	___	___
	Children	Low	2	*Staphylococcus spp. (n=2)*
		High	9	*Staphylococcus spp. (n=3)*
				*Streptococcus spp. (n=1)*
				*Micrococcus spp. (n=1)*
	Adolescents	Low	2	*Escherichia coli (n=2)*
		Standard	2	*Stenotrophomonas (n= 2)*
				*Maltophilia*
		High	4	*Staphylococcus spp. (n=2)*
				*Escherichia coli (n=1)*
				*Veillonella spp. (n=1)*

Among all 330 bacterial isolates, antimicrobial resistance was confined to *Escherichia coli* and *Klebsiella spp*. exhibiting Extended-Spectrum β-lactamase (ESBL) and Carbapenem-Resistant Enterobacterales (CRE) phenotypes. ESBL-producing isolates showed reduced susceptibility to beta-lactams, ciprofloxacin, and trimethoprim–sulfamethoxazole. Among CRE-producing *Klebsiella spp.* isolates, resistance was observed to ertapenem, while susceptibility to imipenem and meropenem was retained. Detailed susceptibility percentages for all antibiotics are presented in Table 4.

**Table 4:** Antimicrobial susceptibility patterns of *Escherichia coli* and *Klebsiella* spp. exhibiting ESBL and CRE phenotypes.

Antibiotic	ESBL¹*Escherichia coli* *(22 isolates)*	CRE² *Escherichia coli(3 isolates)*	ESBL¹ *Klebsiella spp.(14 isolates)*	CRE² *Klebsiella spp.(2 isolates)*
**Ampicillin–Sulbactam**	4/22 (18%)	0/3 (0%)	3/14 (21%)	0/2 (0%)
**Piperacillin–Tazobactam**	12/22 (55%)	0/3 (0%)	9/14 (64%)	0/2 (0%)
**Ticarcillin–Clavulanate**	8/22 (36%)	NA³	NA³	NA³
**Ceftazidime**	0/22 (0%)	0/3 (0%)	0/14 (0%)	0/2 (0%)
**Ceftriaxone**	0/22 (0%)	0/3 (0%)	0/14 (0%)	0/2 (0%)
**Cefepime**	7/22 (32%)	0/3 (0%)	7/14 (50%)	1/2 (50%)
**Imipenem**	22/22 (100%)	0/3 (0%)	12/14 (86%)	2/2 (100%)
**Meropenem**	22/22 (100%)	0/3 (0%)	12/14 (86%)	2/2 (100%)
**Ertapenem**	19/22 (86%)	0/3 (0%)	12/14 (86%)	0/2 (0%)
**Ciprofloxacin**	13/22 (59%)	0/3 (0%)	5/14 (36%)	0/2 (0%)
**Trimethoprim/sulfamethoxazole**	2/22 (9%)	0/3 (0%)	3/14 (21%)	0/2 (0%)
**Gentamicin**	18/22 (82%)	3/3 (100%)	10/14 (71%)	2/2 (100%)
**Aztreonam**	0/22 (0%)	0/3 (0%)	0/14 (0%)	0/2 (0%)
**Amikacin**	22/22 (100%)	1/3 (33%)	14/14 (100%)	2/2 (100%)
**Ceftazidime–Avibactam**	11/22 (50%)	2/3 (67%)	14/14 (100%)	2/2 (100%)
**Colistin**	0/22 (0%)	0/3 (0%)	0/14 (0%)	0/2 (0%)
**Vancomycin**	NA	NA	NA	NA

¹ESBL: Extended-spectrum β-lactamase–producing isolates.

ESBL was determined using phenotypic confirmatory methods in accordance with CLSI recommendations.

²CRE: Carbapenem-resistant Enterobacterales.

CRE were defined phenotypically as Enterobacterales isolates demonstrating non-susceptibility to at least one carbapenem agent (imipenem, meropenem, or ertapenem) according to CLSI interpretive criteria.

³NA indicates that the antibiotic was not tested for that organism group.

Susceptibility percentages represent the proportion of susceptible (S) isolates among those tested.

Intermediate (I) results were not considered susceptible.

Each cultured organism was counted as a separate isolate. In cases of polymicrobial bacteremia, each organism was analyzed independently. Repeated positive blood cultures from the same patient were included as separate isolates.

## Discussion

This study aimed to describe the bacterial profile and antibiotic susceptibility of bloodstream infections among infants, children, and adolescents with acute leukemia across all treatment phases. Our findings summarize descriptively age- and treatment-phase related differences in pathogen distribution, with potential implications for empiric antimicrobial selection and infection-prevention strategies in pediatric oncology.

Across all age groups, *Staphylococcus spp.* were the predominant pathogen, consistent with previously published cohorts of pediatric patients with acute leukemia [6,9]. This predominance is expected given the widespread use of CVC and chemotherapy induced mucositis, which is causing a shift toward Gram-positive coccal bacteremia [17]. However, a higher proportion of Gram-negative pathogens was observed among adolescents, including *Escherichia coli, Klebsiella spp., and Pseudomonas spp.* These findings may be relevant when considering empiric antimicrobial selection in older pediatric age groups, particularly in patients with recurrent admissions, bacteremia episodes, or prolonged neutropenia.

The distribution of pathogens showed descriptive differences across treatment phases. Bacteremia was most frequent during induction and consolidation, which represents the most intensive parts of leukemia therapy. During these phases, patients —particularly those in high-risk groups—receive intensive chemotherapy, have CVCs inserted, and experience treatment-related complications such as profound myelosuppression, febrile neutropenia, and mucositis, often necessitating prolonged hospitalization and increasing the risk of infections from endogenous flora and nosocomial sources. These findings reinforce the importance of close infection monitoring, timely empiric broad-spectrum antimicrobial therapy, and supportive care measures during early intensive treatment phases. Although maintenance therapy is less intensive, it still accounted for a substantial number of episodes because of its prolonged duration of two to three years and the limited neutrophil recovery following steroid pulses [18]. These findings are consistent with previous studies reporting that most bacteremia infections occur during induction and maintenance [6,9].

In contrast, fewer bacteremia episodes were observed during relapse and palliative care. Relatively, more Gram-negative pathogens were identified, particularly among adolescents. However, these observations should be interpreted cautiously given a smaller number of episodes in these treatment phases. Differences in prior antibiotic exposure – with increasing multidrug resistance –, cumulative chemotherapy intensity, and the combined effects of age and treatment intensity may have contributed to the observed distribution of pathogens [19].

In AML, the overall number of bacteremia episodes was lower, which may be partly attributable to the smaller patient population at our center compared with ALL. In addition, the routine use of prophylactic vancomycin and ciprofloxacin when the ANC declines may potentially influence pathogen distribution and infection frequency; however, this study was not designed to evaluate the effect of prophylactic antimicrobial strategies [8,20].

The antimicrobial agents included in susceptibility testing were selected based on institutional microbiology protocols, antibiograms and commonly used empiric and targeted therapies for febrile neutropenia and bloodstream infections in pediatric oncology patients. Attention was given to β-lactams, carbapenems, aminoglycosides, and glycopeptides due to their frequent use in empiric management of high-risk febrile neutropenia. In this study, most antibiotic resistance was observed among ESBL- and CRE-producing *Escherichia coli* and *Klebsiella spp.*, consistent with global trends in multidrug-resistant Enterobacterales [21]. A study by Hassouneh et al. also reported that the highest prevalence of multidrug-resistant pathogens was observed in *Escherichia coli* (45%) and *Klebsiella pneumoniae* (21%) [22]. Surveillance studies from Europe, Asia, and the Middle East have reported a rising prevalence of ESBL- and CRE-producing *Escherichia coli* and *Klebsiella spp.* in both hospital and community settings, driven by broad antibiotic selection and the dissemination of mobile resistance genes. This phenomenon is particularly pronounced in pediatric and immune-suppressed populations, such as children with leukemia, who are frequently exposed to broad-spectrum antibiotics [23].

This study has several limitations. As a retrospective single-center analysis, findings may not be generalizable to other settings with different microbiological profiles or infection-control practices. Contamination was defined solely based on the absence of clinical signs or symptoms, which may have led to misclassification. Clinical outcomes including mortality, and inferential statistical analyses were not examined but represent key areas for future research. Multiple bacteremia episodes from the same patient may also have contributed to repeated observations. Furthermore, the infants, adolescents and AML subgroups were limited in number, restricting the interpretation and generalizability of subgroup-specific findings.

Despite these limitations, this study addresses an important gap by providing an age-stratified assessment of bacteremia patterns across the full course of leukemia treatment and may help inform future studies evaluating age-tailored empiric antimicrobial strategies in pediatric oncology. Future multicenter studies with larger AML, infants, and adolescents cohorts are needed to better characterize age-specific pathogen distribution and resistance patterns in pediatric patients with acute leukemia. Prospective studies evaluating clinical outcomes, recurrent bacteremia episodes, antimicrobial prophylaxis strategies, and the impact of empiric antibiotic selection on resistance trends would further strengthen infection-management approaches in this population.

## Conclusions

Descriptive differences in bacteremia episodes and bacterial isolates were observed across age groups, treatment phases, and risk categories. Larger multicenter studies are needed to determine whether these patterns should inform age- or phase-specific empiric therapy strategies.

## References

[1] Pizzo PA, Rubin M, Freifeld A, Walsh TJ. The child with cancer and infection. I. Empiric therapy for fever and neutropenia, and preventive strategies. J Pediatr. 1991;119(5):679–94. doi: 10.1016/s0022-3476(05)80281-1 1941374

[2] Zajac-Spychala O, Wachowiak J, Gryniewicz-Kwiatkowska O, Gietka A, Dembowska-Baginska B, Semczuk K, et al. Prevalence, epidemiology, etiology, and sensitivity of invasive bacterial infections in pediatric patients undergoing oncological treatment: a multicenter nationwide study. Microb Drug Resist. 2021;27(1):53–63. doi: 10.1089/mdr.2019.0393 32434455

[3] Simon AK, Hollander GA, McMichael A. Evolution of the immune system in humans from infancy to old age. Proc Biol Sci. 2015;282(1821):20143085. doi: 10.1098/rspb.2014.3085 26702035 PMC4707740

[4] Singer CE, Bica EC, Gaman S, Varut RM, Pluta ID, Radulescu V, et al. Age-Stratified clinical and microbiological profiles in pediatric infectious disease admissions: implications for risk prediction and antimicrobial stewardship. Pharmaceutics. 2025;17(11):1472. doi: 10.3390/pharmaceutics17111472 41304809 PMC12655326

[5] Carlesse F, Lopes de Sousa AV. Infections in children and adolescents with Acute Leukemia. EJC Paediatric Oncology. 2024;3:100142. doi: 10.1016/j.ejcped.2024.100142

[6] Katsibardi K, Papadakis V, Charisiadou A, Pangalis A, Polychronopoulou S. Blood stream infections throught the entire course of acute lymphoblastic leukemia treatment. Neoplasma. 2011;58(4):326–30. doi: 10.4149/neo_2011_04_326 21520989

[7] Silva RAM, de Mendonça RMH, Dos Santos Aguiar S, Yajima JC, Marson FAL, Brandalise SR, et al. Induction therapy for acute lymphoblastic leukemia: incidence and risk factors for bloodstream infections. Support Care Cancer. 2022;30(1):695–702. doi: 10.1007/s00520-021-06471-8 34363492

[8] Rogers AEJ, Eisenman KM, Dolan SA, Belderson KM, Zauche JR, Tong S, et al. Risk factors for bacteremia and central line-associated blood stream infections in children with acute myelogenous leukemia: a single-institution report. Pediatr Blood Cancer. 2017;64(3):10.1002/pbc.26254. doi: 10.1002/pbc.26254 27616655

[9] Katsimpardi K, Papadakis V, Pangalis A, Parcharidou A, Panagiotou JP, Soutis M, et al. Infections in a pediatric patient cohort with acute lymphoblastic leukemia during the entire course of treatment. Support Care Cancer. 2006;14(3):277–84. doi: 10.1007/s00520-005-0884-6 16270193

[10] Viscoli C. Bloodstream infections: the peak of the iceberg. Virulence. 2016;7(3):248–51. doi: 10.1080/21505594.2016.1152440 26890622 PMC4871637

[11] St. Jude Children’s Research Hospital. TOTXV: TOTAL Therapy Study XV for Newly Diagnosed Patients with Acute Lymphoblastic Leukemia. Available from: https://www.stjude.org/care-treatment/clinical-trials/totxv-acute-lymphoblastic-leukemia.html

[12] Omar AA, Basiouny L, Elnoby AS, Zaki A, Abouzid M. St. Jude total therapy studies from I to XVII for childhood acute lymphoblastic leukemia: a brief review. J Egypt Natl Canc Inst. 2022;34(1):25. doi: 10.1186/s43046-022-00126-3 35696003 PMC13314260

[13] Parker C, Waters R, Leighton C, Hancock J, Sutton R, Moorman AV, et al. Effect of mitoxantrone on outcome of children with first relapse of acute lymphoblastic leukaemia (ALL R3): an open-label randomised trial. Lancet. 2010;376(9757):2009–17. doi: 10.1016/S0140-6736(10)62002-8 21131038 PMC3010035

[14] Jeha S, Pei D, Choi J, Cheng C, Sandlund JT, Coustan-Smith E, et al. Improved CNS control of childhood acute lymphoblastic leukemia without cranial irradiation: St Jude total therapy study 16. J Clin Oncol. 2019;37(35):3377–91. doi: 10.1200/JCO.19.01692 31657981 PMC7351342

[15] Rubnitz JE, Inaba H, Dahl G, Ribeiro RC, Bowman WP, Taub J, et al. Minimal residual disease-directed therapy for childhood acute myeloid leukaemia: results of the AML02 multicentre trial. Lancet Oncol. 2010;11(6):543–52. doi: 10.1016/S1470-2045(10)70090-5 20451454 PMC3171799

[16] Weinstein MP, Lewis JS 2nd. The clinical and laboratory standards institute subcommittee on antimicrobial susceptibility testing: background, organization, functions, and processes. J Clin Microbiol. 2020;58(3):e01864-19. doi: 10.1128/JCM.01864-19 31915289 PMC7041576

[17] Zinner SH. Changing epidemiology of infections in patients with neutropenia and cancer: emphasis on gram-positive and resistant bacteria. Clin Infect Dis. 1999;29(3):490–4. doi: 10.1086/598620 10530434

[18] Inaba H, Pei D, Wolf J, Howard SC, Hayden RT, Go M, et al. Infection-related complications during treatment for childhood acute lymphoblastic leukemia. Ann Oncol. 2017;28(2):386–92. doi: 10.1093/annonc/mdw557 28426102 PMC5834143

[19] Cattaneo C, Antoniazzi F, Tumbarello M, Skert C, Borlenghi E, Schieppati F, et al. Relapsing bloodstream infections during treatment of acute leukemia. Ann Hematol. 2014;93(5):785–90. doi: 10.1007/s00277-013-1965-0 24288110

[20] Matsukawa Y, Matsubayashi J, Sakamoto K, Takashima K, Ikeda Y, Osawa M, et al. Incidence of bloodstream infections in pediatric patients with cancer during febrile neutropenia: a retrospective study. JMA J. 2025;8(2):560–7. doi: 10.31662/jmaj.2024-0369 40416004 PMC12095455

[21] Breijyeh Z, Jubeh B, Karaman R. Resistance of gram-negative bacteria to current antibacterial agents and approaches to resolve it. Molecules. 2020 Mar 16;25(6):1340. doi: 10.3390/molecules25061340 PMC7144564 32187986

[22] Hassouneh D, Zatarah R, AbuSara A, Nazer L, Abu Ghosh A, Aryan HA, et al. Microbiological profiles and resistance patterns in pediatrics with cancer: an 8-year study at a comprehensive cancer center in Jordan. Cancer Rep (Hoboken). 2025;8(3):e70132. doi: 10.1002/cnr2.70132 40065500 PMC11893483

[23] Karlowsky JA, Lob SH, DeRyke CA, Siddiqui F, Young K, Motyl MR, et al. Prevalence of ESBL non-CRE Escherichia coli and Klebsiella pneumoniae among clinical isolates collected by the SMART global surveillance programme from 2015 to 2019. Int J Antimicrob Agents. 2022;59(3):106535. doi: 10.1016/j.ijantimicag.2022.106535 35091052

